# The Cadaveric Studies and the Definition of the Antero-Lateral Ligament of the Knee: From the Anatomical Features to the Patient-Specific Reconstruction Surgical Techniques

**DOI:** 10.3390/ijerph182312852

**Published:** 2021-12-06

**Authors:** Giacomo Dal Fabbro, Piero Agostinone, Gian Andrea Lucidi, Nicola Pizza, Nicolò Maitan, Alberto Grassi, Stefano Zaffagnini

**Affiliations:** 1 2nd Orthopaedics and Trauma Unit, IRCCS Rizzoli Orthopaedic Institute, 40136 Bologna, Italy; piero.agostinone@studio.unibo.it (P.A.); gianandrea.lucidi@studio.unibo.it (G.A.L.); Nicola.pizza@studio.unibo.it (N.P.); nicolo.maitan@studio.unibo.it (N.M.); alberto.grassi@ior.it (A.G.); stefano.zaffagnini@unibo.it (S.Z.); 2 Dipartimento di Scienze Biomediche e Neuromotorie DIBINEM, University of Bologna, 40126 Bologna, Italy

**Keywords:** human anatomy, cadaver dissection, surgical technologies, sports and anatomy, history of medicine

## Abstract

Cadaver studies represented a milestone in surgical orthopaedic research, and still today they play a crucial role in the achievement of new knowledge about joint disease behaviour and treatment. In this review, an overview of the cadaver studies available in the literature about the anatomy, role, and treatment of the antero-lateral ligament (ALL) of the knee was performed. The aim of the review was to describe and gain more insight into the part of in vitro study in understanding knee joint anatomy and biomechanics, and in developing surgical reconstruction techniques. The findings of the review showed that cadaver studies had, and will continue to have, a key role in the research of knee joint biomechanics and surgical reconstruction. Moreover, they represent a powerful tool to develop and test new devices which could be useful in clinical and surgical practice.

## 1. Introduction

A deep knowledge of human anatomy is mandatory for surgeons, and the possibility of directly studying human corpses surely improves the training of all the medical students. Cadaveric studies are milestones in the orthopaedic research; they permitted our predecessors to investigate the basis of many illnesses and, in orthopaedics, the anatomical description of fundamental structures that could be injured, such as ligaments, bones, and muscles [1].

A further step was the possibility to test the biomechanics of the joints using cadavers. These studies allowed the surgeons to better understand the roles of anatomical structures in restraining joints from pathological movements in order to develop surgical techniques that became more and more accurate, not only anatomically, but also biomechanically.

Although the orthopaedic surgical techniques have improved in leaps and bounds in recent decades, we are still far from the possibility of completely restoring injured anatomical structures. In this regard, one of the most debated topics in knee surgery is the necessity to improve anterior cruciate ligament (ACL) reconstructions because the standards of treatment of this kind of injury, one of the most common not only among athletes, but also common people [2], is far from being considered perfect. One of the main problems in this sense is the presence of residual rotatory instability after ACL reconstruction, an event that severely affected sportive people, but also could explain the higher rate of osteoarthritis among people who underwent this type of surgery [3].

To explain the residual rotatory laxity, the most credited theory is the damage of the secondary knee internal rotation restrainer during the ACL injury, located in the antero-lateral aspect of the knee. This speculation derives from the association of a Segond fracture to an ACL tear, and has recently been confirmed by cadaveric studies [4]. Among them, the anatomical study by Claes et al. [5] is one of the most cited because the author led the researchers to rediscover the antero-lateral structures of the knee.

The possible involvement of lateral knee structures in an ACL injury is the anatomical base of lateral extra-articular tenodesis (LET), additional procedures performed in patients with a higher risk of residual laxity after ACL reconstruction surgery [6]. Nowadays, several different LETs have been developed, some to reproduce a hypothetical damaged anatomical structure such as the antero-lateral ligament (ALL), others, not anatomic, to biomechanically “reinforce” the lateral side of the knee [7]. Testing in vitro the role of the LET procedure in knee stability is mandatory to investigate that the techniques are safe and to identify differences among them.

The aim of the present study is to perform a narrative review of the in vitro studies involved in the anatomical description, biomechanical analysis of the role and the effect of different LET procedures on the knee joint behaviour, and to demonstrate how the cadaveric investigations have an essential role in developing new surgical knowledge and techniques, possibly targeted to specific patients.

## 2. Methods

### 2.1. Search Strategy

The literature research was performed in relevant databases (MEDLINE, EMBASE and Google Scholar) and through reference checking, manual searching in relevant journals and expert recommendations. The research was performed for anatomical and biomechanical human cadaveric studies regarding the feature of the antero-lateral ligament (ALL) of the knee, the effect of its rupture and the in vitro biomechanical results of the reconstruction technique in the setting of ACL-injured and ACL-reconstructed knee.

### 2.2. Data Analysis

Due to the diversity of studies and heterogeneity of methods and parameters measures, a narrative review was considered the most appropriate way to review the literature.

To underline the significant role of the cadaveric studies in developing orthopaedic surgical treatment, we divided the included studies into three groups:

- the anatomical studies, about the discovery and description of the anatomical features of the ALL
- the cutting studies, in which the biomechanical and kinematic effect of the ALL rupture was investigated
- the reconstruction studies, in which the results regarding the knee behaviours after different kinds of knee lateral extra-articular techniques, both isolated or associated with ACL reconstruction, were compared.

## 3. Results

### 3.1. The Anatomical Studies: How Is It Like?

The first historical reference about the anatomical description of the ALL in cadavers was provided by the German anatomist Josias Weitbrecht in 1752, who described in the knee “fibrous bunches that reinforce the capsule and bands that supplement the fixation of semicircular cartilage (the meniscus)”. In 1879 the French surgeon Paul Segond [8] described a remarkably constant avulsion fracture pattern at the antero-lateral proximal tibia as a result of forced internal rotation of the knee This eponymous Segond fracture was reported to occur in the tibial region “above and behind the tubercle of Gerdy”. Moreover, during an autopsy observation, Segond highlighted a “pearly, resistant fibrous band” located in the antero-lateral aspect of the knee, which is now advocated to be the ALL [9]. However, in both these historical works, no precise anatomical description was provided. Around 1920, French anatomists Vallois [10] and Jost [11] expanded the anatomic description by reporting a structure “arising from the lateral femoral epicondyle. Through an oblique anteroinferior course, and after getting slightly wider, it attaches to the superior and peripheral edge of anterior horn of the lateral meniscus [...]. It ends at the tibia”.

Moving to the modern era of knee ligament surgery and modern cadaveric studies, Terry and his colleagues described, in 1993, an interconnection between the anterior cruciate ligament (ACL) and the capsulo-osseous layer of the ileo-tibial band (ITB), running from the femoral epicondylar region to the proximal lateral tibia [12].

In their anatomical study of 2013, Claes et al. used 41 embalmed cadaveric knees, which underwent dissection of the antero-lateral compartment of the knee, to perform a qualitative and quantitative characterisation of the ALL [5]. In the latter study, each ALL was described with regard to origin, insertion, relationship with nearby anatomical structures such as lateral collateral ligament, lateral meniscus, lateral intermuscular septum, Gerdy’s tubercle, and the tip of the femoral head. To standardise the measurements, the authors put the tibia of all included cadavers in a reduced position with respect to the femur, with the foot in neutral rotation. The results showed in all but one of the 41 dissected knees a distinct ligamentous structure, easily distinguishable from the inner joint capsule lying anterior to it, located in the antero-lateral side of the knee joint, connecting the femur with the tibia. These findings have been supported by many following anatomic studies which have reported the incidence of the ALL to be between 50% and 100% in cadaveric specimens drawn from several populations [9].

Several studies followed the one by Claes and his colleagues, providing results that often contrasted [13,14,15]. In particular, the main issue regarded the ALL femoral insertion and its relationship with the lateral meniscus. In a descriptive laboratory study with thirteen unpaired knees, Helito et al. [16] described two different structures among the ALL and defined it as its deep and the superficial layer.

**Figure 1:**
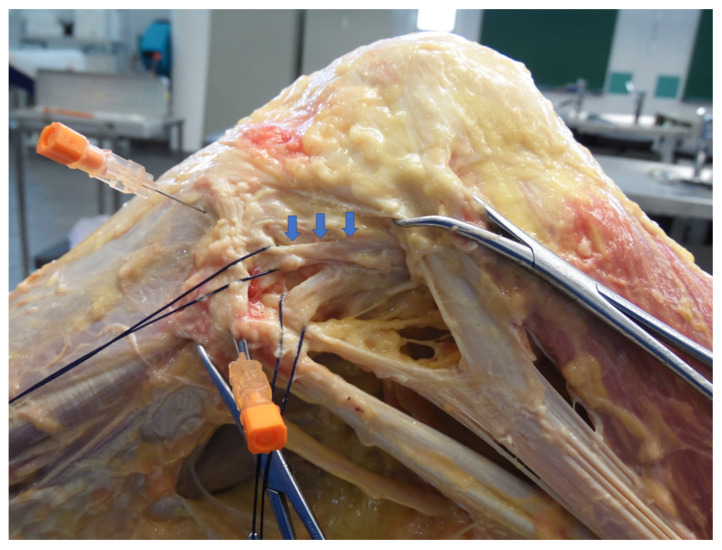
Dissection and isolation of the ALL (blue arrow) in a fresh-frozen cadaveric knee.

Most of these dissection-based cadaveric studies have employed fresh frozen cadavers with several different dissection strategies and procedures [13,14,17] (Figure 1). Dodds et al. performed on 40 fresh-frozen knees a dissection of the superficial antero-lateral structure, elevating the iliotibial band from the posterior to anterior and peeling off the Gordy’s tubercle. After examining the antero-lateral structures from the superficial aspect, the authors opened and disarticulated the knees from the medial side with only the antero-lateral structures remaining intact, with the purpose of facilitating the examination of their deep aspect with the transillumination to display the capsular thickness [13].

In a dissection-based study, in which embalmed cadavers were used [18], Parker et al. described and assessed the feasibility of a new method for dissecting the ALL ligament: the authors, in a different way compared to previous studies with embalmed cadavers [5,19], performed an additional cut to transversely incise the quadriceps femoris tendon, with the aim to obtain full mobility of the knee joint, making the LCL and the ALL clearly identifiable. The results of the latter study showed that, using this dissection technique, the ALL appeared to be clearly distinguishable from the joint capsule. Moreover, the authors associated the absence of the ALL ligament, reported in two specimens out of 53 included in the study, with the unique superficial presence of the lateral inferior genicular vessels upon dissection.

Among the cadaveric studies about the ALL present in the literature, besides the classical anatomical dissection, further methods of analysis have been used to better define the features of this knee structure with the information that could be obtained from the specimens. Caterine et al. [17] performed a controlled laboratory study in which ten fresh-frozen cadaveric knees underwent 3 T magnetic resonance imaging (MRI) before the anatomical dissection was performed. The authors reported that the ALL could be identified as a distinct, distinguishable structure in all 10 specimens using the MRI [17]. In another cadaver study, Zappia et al. performed a high resolution, real time ultrasonography evaluation of the ALL in eight specimens [20]; the results of the latter study showed that the ALL was distinguished in all eight cadaver limbs, and that the ultrasound examination was a useful tool to investigate the ALL features and its injuries.

### 3.2. The Cutting Studies: How It Works?

The anatomical rediscovery of the ALL renewed the interest in its function and biomechanical role. In particular, the ALL’s role as a secondary restraint of the knee and as a key factor in the ACL reconstruction failure has been emphasised in literature. The cadaver cutting studies played a crucial role in the analysis of the stabilising function of the ALL in the knee with an associated ACL rupture, which is well known as the primary restraint to the tibial anterior translation. The protocol of these studies consists of progressive resections of the knee restraint structures, starting from the ACL, the primary knee restraint, and arriving at the ALL, the secondary knee restraint. In the majority of studies, the biomechanical analysis was performed with regard to the kinematics of the knee with and without the associated ALL resection.

The assessment of the changes in ACL deficient knee biomechanics after ALL resection has been conducted with different kinds of tools among distinct cadaver studies [21].

In several cutting studies, a six degree of freedom robotic setup has been used. Rasmussen et al., using this kind of cadaver setup, reported an increase in knee internal rotation when the ALL was cut [4]. In another cadaveric study, Pearson et al. found a significant contribution of the ALL in restraining internal rotation at 30–90° of knee flexion under a 5 nM moment load [22]. Moreover, the cadaver biomechanical analysis allows the use of other measurement tools, often with the aim to develop and reproduce the using of systems and devices available in the in vivo clinical practice [23].

**Figure 2:**
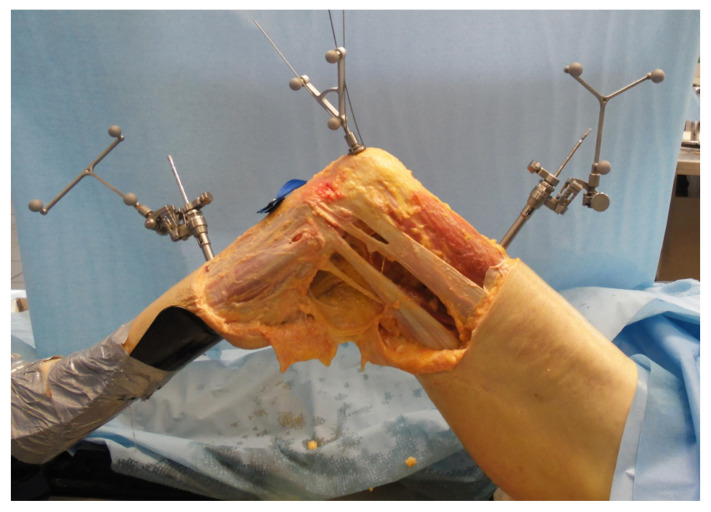
Setting of a cadaveric investigation of knee kinematics using a surgical navigation system.

**Figure 3:**
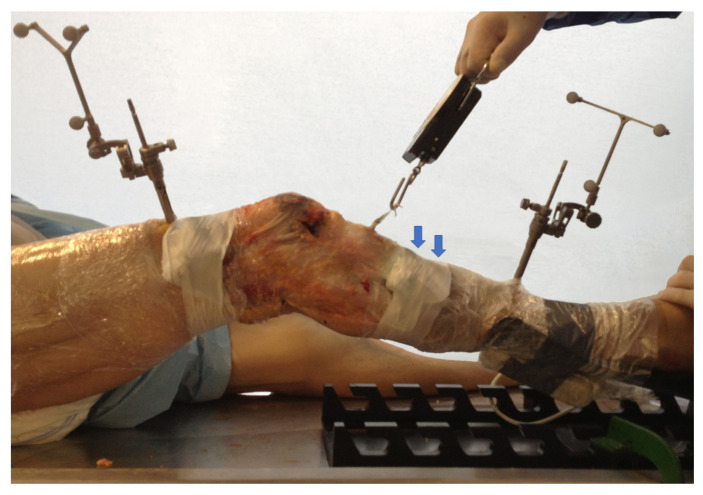
Setting of a cadaveric investigation using non-invasive inertial sensor (blue arrows).

Bonanzinga et al. [24] analysed the joint laxity of 10 fresh-frozen knees in three different conditions: intact, ACL-deficient, and associated ACL and ALL resected. In this study, the authors assessed the kinematics of the knee using a surgical navigation system (Figure 2). The results showed that the ALL plays a significant role in controlling rotatory knee laxity during both static and dynamic evaluation. On the other hand, the ALL resection did not produce a significant increase in terms of anterior displacement. In line with these findings, Grassi et al. [25] reported an increased rotatory laxity in the ACL deficient knees, associated with ALL lesions compared with only ACL–injured knees. In the latter study, the authors performed the biomechanical test in ten fresh-frozen cadaveric knees using a non-invasive skin-fixed inertial sensor (Figure 3).

### 3.3. The Reconstruction Studies: How to Restore It?

Since the ALL has been showed to play a significant role as the secondary restraint of the rotatory laxity of the knee, a different type of antero-lateral reconstruction technique, in association with the ACL reconstruction, has been described and tested among in vitro studies [7]. The aim of the several extra-articular antero-lateral procedures associated with ACL reconstruction introduced into the literature was to improve the control of the knee rotational laxity over an isolated intra-articular procedure [26]. Therefore, the majority of biomechanical studies about this issue compared the differences in the knee kinematic restoration between the isolated intra-articular ACL reconstruction techniques, and procedures in which a lateral extra-articular reconstruction was associated with the intra-articular ACL reconstruction.

Bonanzinga et al., using a surgical navigation system, reported more efficient control of internal knee rotation in ten fresh-frozen knees by the associated intra-articular and antero-lateral reconstruction, than by an isolated intra-articular technique [27].

Neri et al., in a recent in vitro study, compared the ability of a variety of antero-lateral procedures [28] in association with ACL reconstruction, to restore the native knee kinematics. The authors tested the kinematic effects of the five different antero-lateral procedures in ten fresh-frozen cadavers using a motion analysis 3-D optoelectronic system, and distinguished two main kinds of extra-articular antero-lateral procedures: the antero-lateral ligament reconstruction and the lateral tenodesis using a section of ileo-tibial band (ITB). Results showed that the antero-lateral tenodesis with the ITB achieved excellent rotational control, but over-constrained the internal rotation in non-physiologic kinematics compared to the antero-lateral ligament reconstruction.

Moreover, the cadaver studies allowed the analysis of the changes of the mechanics of both the intra-articular and the extra-articular reconstruction, before and after the addition of an extra-articular antero-lateral procedure. Engebretsen et al. [29], analysing seven fresh-frozen knees, reported a significant decrease in the total intra-articular graft force experienced by the reconstructed ACL over all the four tested flexion angles, after the addition of a lateral extra-articular procedure. The authors of the latter study assessed the force experienced by the intra-articular graft with a buckle transducer, consisting of a crossbar and a frame with two semiconductor strain gages. In another in vitro study, Draganich et al. [30], using a strain gauge, assessed the strain pattern showed by an extra-articular antero-lateral reconstruction in an ACL deficient status from 0° to 90° of knee flexion. The results showed that the antero-lateral reconstruction increased anterior and rotatory stability between 30° and 90° of flexion.

## 4. Discussion

The main finding of the current review is that, despite several cadaveric studies that investigate this issue, the anatomy, the function and the best surgical reconstruction technique of the antero-lateral ligament (ALL) of the knee remain still not fully known. The main reason for this is represented by the confusing terminology, the different dissection techniques, and specimen characteristics used among these studies. On the other side, the results of the current review showed that cadaver studies are the most important tools to gain more insight into the features of the ALL, and to better understand it’s role in the biomechanics of the knee joint.

In 2013, Claes and his colleagues published an anatomical study that is, even today, considered a landmark that renewed one of the principal controversies of the orthopaedic community: the existence, the anatomy and the role of the so-called antero-lateral ligament of the knee (ALL).

Since the precursor of historical studies, in which this knee joint feature was described, among which the most famous and cited is the Paul Segond one [8], the analysis of the knee cadaveric specimen features and the assessment of the behaviour of the corpse joints and tissue in response to external mechanical input, represented a key tool to understanding the real structure and the function of the ALL. Furthermore, after the modern definition of this knee ligament [5], cadaver studies became one of the main ways to develop and test different kinds of surgical reconstruction of the ALL [28].

From this narrative review of the literature, it is clear that the cadaver studies played a striking role in the most significant debate with regard to the ALL, the one which surrounds its anatomy. Further to the study of Claes and his colleagues, several cadaver analyses were performed with the aim to test the results and discuss the conclusion reported by that paper.

The 2017 consensus paper from the ALL expert group [31], defined the ALL as a distinct ligament of the antero-lateral aspect of the human knee, on the basis of its reported incidence among the cadaver studies, which resulted between the 50% and 100% [9].

Among these anatomical studies, different in vitro set ups have been used: embalmed or fresh-frozen specimens, and various dissection techniques and assessment tools. Parker et al. [18] proposed a new dissection technique to achieve a fuller exposure of the ALL with regard to the joint capsule. The results of the current study strengthen the concept of the ALL as a discrete ligamentous structure. However, a full consensus about the anatomical features of the ALL still lacks, in particular with regard to the ALL femoral insertion and its relationship with the lateral meniscus [13,14,15]. Helito et al. [16], in their cadaver study, proposed an intriguing theory of the two layers of the ALL, which may be consistent with the previous but conflicting cadaveric description of the ALL. However, with the low number of specimens involved, the conclusions of the work of Helito et al. needs further anatomical studies to be confirmed.

Moreover, the current review showed that cadaver studies played a key role also in the analysis of the function of the ALL in the setting of knee joint biomechanics. The cadaveric set up allowed the use of several techniques to assess the effect of the presence and the cutting of the ALL on the knee joint kinematics [21]. A six degree of freedom robotic setup resulted in the most used tools to perform the biomechanical analysis in the cutting studies about the ALL’s role in the joint knee. In some studies, the in vitro analysis was also used to test and validate assessment tools for the knee joint laxity evaluation, which are also available in the clinical practice, such as surgical navigation systems and triaxial accelerometers [24], with the aims to recognise a patient specific injury pattern and address the injury with a customised treatment. Results reported by the cadaver cutting studies showed uniformly that the antero-lateral ligament represent a main secondary restraint of the knee, in particular with regard to limitation of the rotatory knee joint laxity in the setting of the ACL deficient and reconstructed knee [4,21,22,23,24,25].

The accepted biomechanical role of the ALL, supported by the results of cadaver biomechanical studies, led to the addition of lateral extra-articular procedures, with the aim to improve the rotational knee stability in the setting of the ACL reconstruction surgery [26]. The current reviews of literature showed the significant role of the cadaver studies in the development and testing of several surgical procedures for the ALL and rotatory laxity restoration described in the literature. Most of these studies investigated differences in knee kinematics between an isolated ACL reconstruction and an ACL reconstruction associated with an extra-articular lateral plasty. Results from these cadaver studies showed a better rotatory control of the ACL reconstruction coupled with an extra-articular lateral procedure, compared to an isolated ACL reconstruction [27,28]. Some authors reported a non-physiological overstrain of the lateral compartment in patient who underwent associated lateral procedures [28]. However, these findings did not affect clinical outcomes in the in vivo studies at a long term follow up [32]. The analysis on cadavers allowed one also to assess the biomechanical behaviour of the ALL reconstruction among the range of motion [29], and the effects of the addition of an extra-articular lateral procedure on an intra-articular ACL graft force [30]. The effect of this behaviour in an in vivo setting and on the clinical results is still unclear in the literature. In the authors’ clinical experience, the addition of an extra-articular lateral tenodesis in the setting of the ACL reconstruction led to very satisfactory clinical outcomes [32]. However, further both cadaver and in vivo studies are needed to strengthen this evidence.

The main limitation of the current review is that it is a narrative review and it has a less evidence compared with a systematic review design. Moreover, this kind of review does not allow for meta-analysis. However, due to the diversity of studies, and the heterogeneity of methods and parameter measures, a narrative review was considered the most appropriate way to review the literature.

## 5. Conclusions

Cadaver studies played a central role in the discover and definition of the ALL anatomical features, biomechanical role and reconstruction techniques. Further cadaveric investigations are needed to gain more insight into the treatments of an ACL deficient knee and to improve the clinical results of the ACL-injured patient.
